# The fate of Müller’s glia following experimental retinal detachment: nuclear migration, cell division, and subretinal glial scar formation

**Published:** 2010-07-15

**Authors:** Geoffrey P. Lewis, Ethan A. Chapin, Gabriel Luna, Kenneth A. Linberg, Steven K. Fisher

**Affiliations:** Neuroscience Research Institute, University of California, Santa Barbara, CA

## Abstract

**Purpose:**

To study the fate of Müller’s glia following experimental retinal detachment, using a “pulse/chase” paradigm of bromodeoxyuridine (BrdU) labeling for the purpose of understanding the role of Müller cell division in subretinal scar formation.

**Methods:**

Experimental retinal detachments were created in pigmented rabbit eyes, and 3 days later 10 µg of BrdU was injected intravitreally. The retinas were harvested 4 h after the BrdU was administered (i.e., day 3) or on days 4, 7, and 21 post detachment. The tissue was fixed, embedded in agarose, and sectioned at 100 µm. The sections were labeled with various combinations of probes, including anti-vimentin and anti-S100 (as markers for Müller cells), anti-BrdU, anti-phosphohistone H3 (to identify mitotic cells), and the isolectin B4 (to identify macrophages and microglia). Images were captured using an Olympus Fluoview 500 confocal microscope. To aid in our understanding of how Müller cell nuclei undergo cell division, two additional procedures were used: 1) electron microscopy of normal cat and rabbit retinas and 2) a new method using 5-fluorouracil and subsequent anti-BrdU labeling to detect all Müller cell nuclei, using confocal imaging.

**Results:**

Three days after detachment, anti-vimentin labeled all Müller cells, some of which were also labeled with anti-BrdU. On day 4, many of the anti-BrdU-labeled Müller cell nuclei appeared in columns with one labeled nucleus in the inner nuclear layer and another directly sclerad to it in the outer nuclear layer. By day 7, most anti-BrdU-labeled nuclei were observed in subretinal scars. At 3 weeks, some anti-BrdU-labeled nuclei that remained within the retina did not express vimentin or S100. Anti-phosphohistone H3-labeled (i.e., mitotic) cells, some of which were also labeled with anti-BrdU, were only observed in the outer nuclear layer on day 4, and these nuclei were surrounded by an accumulation of vimentin filaments. Isolectin B4-labeled microglia and macrophages also incorporated BrdU and were observed throughout the retina and in subretinal scars during all times of detachment. Electron microscopy and immunofluorescence labeling of the 5-fluorouracil-injected eyes revealed the presence of a unique structural relationship between Müller cell nuclei and intermediate filament proteins.

**Conclusions:**

Following retinal detachment, many Müller cell nuclei initially migrate to the outer retina, undergo mitosis, and eventually reside in subretinal glial scars, suggesting a possible link between the early division of Müller cells and the process of subretinal gliosis. In addition, a subpopulation of anti-BrdU-labeled cells, presumably once Müller cells, appears to stop expressing well accepted Müller cell marker proteins, suggesting a potential dedifferentiation of some of these cells over time. Additionally, Müller cell nuclei may use intermediate filaments as a “track” for migration into the outer retina and later as an important component of cell division by the accumulation of vimentin filaments around the mitotic nuclei.

## Introduction

Injury to the retina, as elsewhere in the central nervous system (CNS), results in the activation of glial cells and the formation of glial scars [1,2]. In retinal detachment (RD), a form of traumatic injury where the retina becomes separated from the underlying retinal pigment epithelial (RPE) layer, the Müller cell, generally considered a specialized radial astrocyte, is the predominant glial cell type involved. Following RD, Müller cells actively proliferate and hypertrophy within the retina and onto either retinal surface where they form structures similar to those formed by reactive astrocytes in the brain and spinal cord [3]. Such cellular scars or “membranes” present on the subretinal or epiretinal (vitreal) surface are considered part of the spectrum of “fibrocontractive retinal disorder” termed proliferative vitreoretinopathy (*PVR*) [4]. Despite numerous attempts to reduce the incidence of PVR, no effective pharmacological treatment has been found to date [5,6]. This may be because PVR is a complex disease involving cell proliferation, growth, spreading, and contractility. Dividing cells have been observed in membranes removed from patients with PVR ([7–9] and unpublished data); however, data from animal models as well as from membranes removed from human patients clearly show a role for Müller cell expansion as a critical part of the response.

Müller cell reactivity can ultimately result in loss of vision because of glial scars on the retinal surfaces. When scars are present in the subretinal space, the regeneration of photoreceptor outer segments is prevented [10]. Subretinal glial scars can also cause visual distortion by preventing proper flattening of the retina or causing macular wrinkling, both of which may require surgical removal of the scar tissue [11]. This is not an insignificant problem since subretinal glial scars in humans have been estimated to occur in 15.7% of all human retinal detachments [12]. When a glial scar grows on the vitreal surface of the retina, the cells can contract, causing retinal folds and re-detachment of the retina. Indeed, epiretinal membrane formation and subsequent contraction detachment remains the most common reason for failure of retinal reattachment surgery and occurs in 5%–12% of cases [13–17]. Although advances in surgical management have improved the ability to ultimately reattach the retina after the occurrence of a contraction detachment, the visual prognosis remains poor, with only 11%–25% of patients achieving a visual acuity of 20/100 [18]. Glial scar formation, therefore, is a serious complication of retinal injury. However, the early events in scar formation are poorly understood.

PVR is generally thought to be a condition involving uncontrolled cell proliferation, although the exact role of proliferation and how it relates to the hypertrophy of Müller cells and ultimate glial scar formation in the injured retina is unclear. It has been demonstrated that within a few hours of detachment, Müller cells upregulate transcription factors related to both proliferation and hypertrophy [19], and at one day after detachment, both events are underway, as evidenced by anti-Ki67 and anti-glial fibrillary acidic protein (GFAP) labeling, respectively [20,21]. Proliferation peaks 3–4 days after detachment, is reduced at day 7, but continues at low levels for as long as the retina remains detached [22,23]. Hypertrophy and growth of Müller cells into the subretinal space also appear to occur as an ongoing event since longer detachment durations result in larger subretinal glial scars.

The question remains, however, as to the role, if any, cellular proliferation has in the formation of glial scars. That is, are glial scars formed only by the population of cells that proliferate? Use of the anti-proliferative agent 5-fluorouracil (5-FU) did not significantly reduce the occurrence of PVR in human patients [6], although issues of timing and dosage make these results difficult to interpret. Moreover, the process of cell division of Müller cells, complex cells that span the entire width of the retina, has not been elucidated in the mammalian retina. To address these issues, we used an intraocular pulse of bromodeoxyuridine (BrdU), given at the peak of the proliferative response after retinal detachment in the rabbit, and followed the distribution of the labeled nuclei over time. We show here that the Müller cell nuclei that incorporate BrdU on day 3 appear to migrate into the outer retina where they undergo mitosis. Some Müller cells with anti-BrdU-labeled nuclei then grow beyond the outer limiting membrane (OLM) to begin the process of glial scar formation. The glial scars expand with increasing detachment time, and many anti-BrdU-labeled nuclei come to reside in the scars. These data suggest a significant role for Müller cell proliferation, beginning early after detachment, in contributing to the formation of subretinal glial scars. In addition, immunocytochemical observations of anti-GFAP and anti-vimentin labeling along with electron microscopic data suggest a link between the migration of Müller cell nuclei and the intermediate filament cytoskeleton.

## Methods

### Retinal detachment surgery

Retinal detachments were created in adult New Zealand Red pigmented rabbits (Myrtle’s Rabbitry, Thompsons Station, TN) by infusing a solution of sodium hyaluronate (Healon, 0.25% in balanced salt solution; Pharmacia, Piscataway, NJ) between the neural retina and RPE via a fine glass pipette. The Healon is necessary to prevent spontaneous reattachment of the retina. The pipette was inserted into the eye via an incision that was made several millimeters below the pars plana to prevent the pipette from touching the lens. Approximately one-half of the inferior retina was detached in the right eye, leaving the superior attached regions as internal controls. The left eyes served as non-detached controls. On day 3 after detachment, the eyes were injected with 10 µg BrdU (Sigma, St Louis, MO) in 50 µl balanced salt solution (BSS; Alcon, Ft. Worth, TX). Animals were euthanized either 4 h after the BrdU injection on day 3, or on days 4, 7, or 21 post detachment, using sodium pentobarbital (120 mg/ml, IV). Three animals were used at each time point. Following enucleation, the eye was fixed in 4% paraformaldehyde for at least 24 h (in 0.1 M sodium cacodylate buffer, pH 7.4; Electron Microscopy Sciences, Fort Washington, PA) and stored until used. All experimental procedures conformed to the ARVO statement for the Use of Animals in Ophthalmic and Vision Research, the Guide for the Care and Use of Laboratory Animals, and the guidelines of the Animal Resource Center of the University of California, Santa Barbara.

### Immunocytochemistry

Following at least 24 h of fixation, retinal pieces approximately 4 mm square, were excised from three detached regions within each eye. The tissue was rinsed in PBS (10X PBS stock: 250 mls distilled water, 21.92 g NaCl, 0.67 g NaH_2_PO_4_, 2.88 g NaH_2_PO_4_; stir well, dilute 1:10 in distilled water, adjust pH to 7.4. Yields 0.086M PBS), embedded in low-melt agarose (5%; Sigma), and sectioned at 100 µm using a vibratome (Technical Products International, Polysciences, Warrington, PA). Sections were incubated in normal donkey serum (1:20) in PBS, 0.5% BSA, 0.1% Triton X-100, and 0.1% azide (PBS, BSA [PBTA]) overnight at 4 °C on a rotator. The following day the sections were pretreated with 2 N HCL for 1 h as an antigen retrieval step for the BrdU. After rinsing in PBTA, the primary antibodies and lectin were added and incubated overnight at 4 °C on a rotator in PBTA. Anti-BrdU (1:200; Accurate Chemical and Scientific Corp., Westbury, NY) was used to detect cells in S-phase; anti-phosphohistone H3 (1:100; Lake Placid, NY) was used to detect cells undergoing mitosis; anti-vimentin (1:500; Dako, Carpinteria, CA) and anti-S100 (1:1000; Dako) were used to identify Müller cells; and isolectin B4, *Griffonia simplicifolia* (1:50; Vector Labs, Burlingame, CA), was used to label microglia and macrophages. Following rinsing of the primary antibodies in PBTA, the secondary probes (streptavidin CY5, donkey antirat CY3, and donkey antimouse CY2; Jackson ImmunoResearch, West Grove, PA) were added together, each at 1:200 in PBTA, overnight at 4 °C on a rotator. On the final day, the sections were rinsed in PBTA, mounted on glass slides, using 5% n-propyl gallate in glycerol, and viewed on an Olympus Fluoview 500 laser scanning confocal microscope (Center Valley, PA). Each image represents a projection from a z-stack of 6–10 images collected at 0.5-µm increments.

### Müller cell nuclei and the cytoskeleton

To systematically study the relationship between intermediate filaments and Müller cell nuclei, we took advantage of the observation that Müller cells appear to specifically take-up 5-FU, which can then be detected with anti-BrdU. Since uracil is normally converted to uridine by cells, presumably the same process occurs with 5-FU after injection into the vitreous, and the product is then detected by anti-BrdU. Why this appears to preferentially label Müller cells remains a mystery but may be related to Müller cells’ close apposition to the vitreous cavity, the site of 5-FU deposition. In this experiment, 5-FU (0.5 mg in 50-µl balanced salt solution) was injected intravitreally into “normal” rabbit eyes (n=3). Three days later animals were euthanized (120 mg/kg Euthasol, intravenous, Henry Schein, Melville, NY), and the eyes fixed as described above. The retinas were then sectioned and labeled with anti-BrdU as well as anti- GFAP (1:400; Dako) and/or anti-vimentin (1:500; Dako), as described below.

Electron microscopy of archived cat and rabbit tissue was also examined to study the ultrastructure of Müller cell nuclei. Both nondetached cat and rabbit retinas and cat retinas detached for 50 days were fixed in 1% glutaraldehyde plus 1% paraformaldehyde for 24 h after which the tissue was rinsed in isotonic phosphate buffer, postfixed for 2 h in 2% osmium tetroxide in phosphate buffer, dehydrated in a graded ethanol series, processed through propylene oxide, and embedded in Spurr’s resin. The retinas were then sectioned at 90 nm in thickness, stained with uranyl acetate and lead citrate, and viewed using a JEOL 1230 electron microscope.

## Results

### Fate of injected bromodeoxyuridine

**Figure 1:**
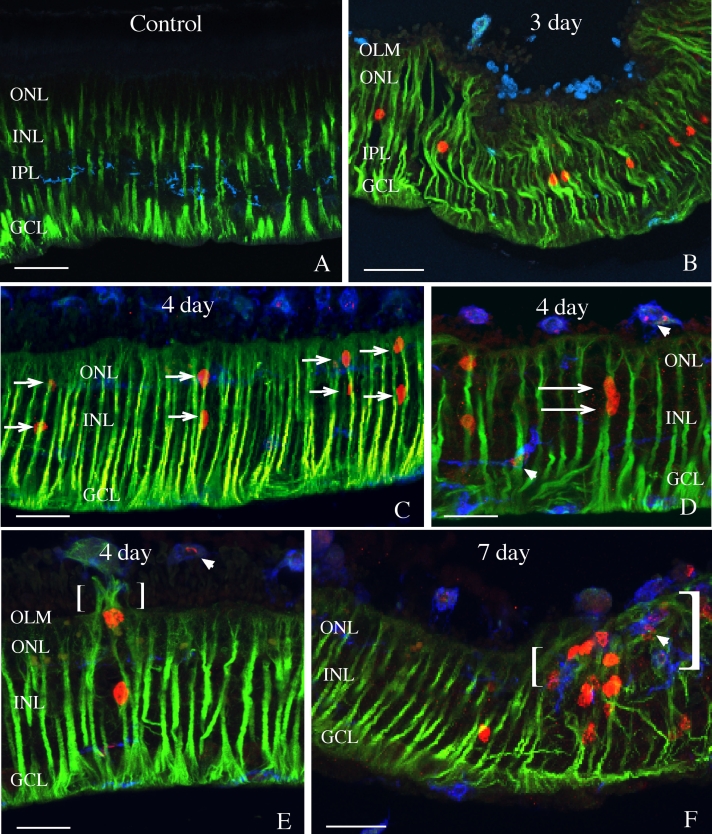
Laser scanning confocal images of control (**A**) and detached (**B**-**F**) rabbit retinas labeled with anti- bromodeoxyuridine (BrdU; red), anti-vimentin (green), and isolectin B4 (blue). BrdU was injected intravitreally on day 3. In control retina (**A**; 4 h after BrdU injection into the normal eye), no BrdU is detected and anti-vimentin labeling of Müller cells extends from their endfeet into the outer nuclear layer (ONL). At 3 days after detachment (4 h after intravitreal injection of BrdU), anti-BrdU labeling is present in many Müller cell nuclei in the inner nuclear layer (INL), and anti-vimentin labeling in Müller cells spans the entire width of the retina. At 4 days after detachment (24 h after BrdU injection), BrdU-labeled nuclei frequently appear in radial columns across the retina (**C**, arrows). In some cases, two nuclei can be observed directly adjacent to one another (**D**, arrows). Anti-BrdU-labeled nuclei are also observed directly adjacent to anti-vimentin-labeled Müller cell processes extending into the subretinal space (**E**, brackets). At 7 days after detachment, many anti-BrdU-labeled Müller cells are observed in large subretinal glial scars that are also labeled with anti-vimentin (**F**, brackets). The isolectin B4 labels the stellate processes of the microglia in the inner plexiform layer (IPL) in the control retina (**A**), but after detachment these cells round-up and migrate throughout the retina and into subretinal glial scars (**B**-**F**). The isolectin B4 also labels macrophages in the subretinal space (**B**-**F**). Some macrophages (in the subretinal space) and microglia (in the retina) are labeled with anti-BrdU (**D**, **E**, **F**, arrowheads). Abbreviations: GCL represents ganglion cell layer; OLM represents outer limiting membrane. Scale bars are equal to 50 µm.

In control nondetached rabbit retinas, anti-vimentin labeling of the Müller cells extends from the inner limiting membrane (ILM) into the outer nuclear layer (ONL; Figure 1A, green). Based on previous data showing that intraretinal proliferation peaks at 3 days in the rabbit [20], BrdU was injected into the vitreous at this time and the tissue examined 4 h later. At this time point, anti-vimentin labeling increased, filling the entire Müller cell from the ILM to the OLM (Figure 1B). Numerous anti-BrdU-labeled Müller cell nuclei could also be seen in the inner nuclear layer (INL; Figure 1B, red). At day 4, 24 h after BrdU injection, labeled nuclei frequently appeared in radial “columns” across the retina, such that one labeled nucleus in the ONL directly overlies another in the INL (Figure 1C, arrows). In some cases two nuclei could be observed directly adjacent to one another, always in a “vertical” arrangement, giving the impression that they occur in the same Müller cell (Figure 1D, arrows). We never observed two labeled nuclei lying “side-by-side” in the INL. Also at the 4-day detachment time, the first sign of Müller cell growth beyond the OLM into the subretinal space was observed (Figure 1E, bracket). Invariably, there was an anti-BrdU-labeled nucleus associated with this early stage of growth, itself often appearing to extend partially beyond the OLM. At 7 days following detachment, many anti-BrdU-labeled nuclei were present within, or adjacent to, large subretinal glial scars (Figure 1F, brackets), although in regions away from the scars, the retina appeared mostly devoid of labeled nuclei (Figure 1F, left half of the image). Subretinal scars, however, were never observed without containing anti-BrdU-labeled nuclei.

Microglia and macrophages also undergo cell division in the retina after detachment [22]. To ascertain that the majority of BrdU labeling was occurring in Müller cells, we used the isolectin B4 to identify the former cell types. As shown previously, the cells within the retina are primarily resident microglia, whereas those in the subretinal space are mostly macrophages that have entered the eye from the blood [24]. Isolectin B4-labeled microglia in attached regions from an eye with a retinal detachment appear as an array of fine stellate processes confined primarily to the inner plexiform layer (IPL; Figure 1A, blue). Following detachment, the microglia round-up and migrate into the outer retina, whereas macrophages appear to remain in the subretinal space (Figure 1B-F; also see ref. #26, for quantitative data). Some macrophages (in the subretinal space) and microglia (in the retina) can be observed labeled with anti-BrdU (Figure 1D-F, arrowheads). In comparison to Müller cells, the number of anti-BrdU-labeled immune cells is very low (Figure 1D). That, combined with the prominent anti-vimentin labeling of Müller cells makes their confusion unlikely. Some retinal regions were relatively devoid of immune cells, while others contained many, but there was no apparent correlation between the presence of these cells and the extent of glial scar formation.

**Figure 2:**
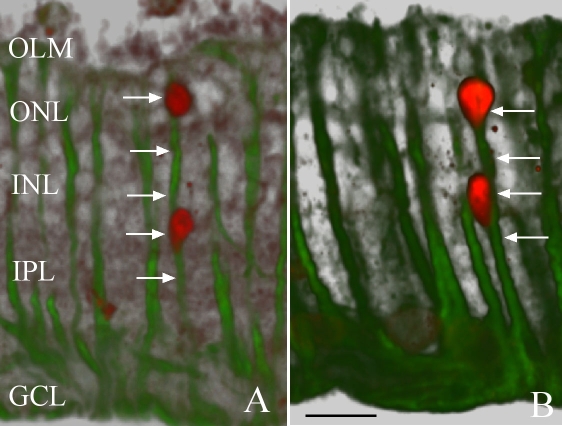
Laser scanning confocal images of rabbit retinas detached for 4 days labeled with anti-bromodeoxyuridine (BrdU; red) and anti-vimentin (green). The z-stack of images is projected in three dimensions at varying contrasts, illustrating that the BrdU-labeled nuclei appear to be associated with only one Müller cell process. **A** shows two nuclei lined up directly with a single Müller cell “stalk” of intermediate filaments (arrows). The image in **B** is rotated slightly so that the lower nucleus in the INL appears off to the side of the major intermediate filament bundle. Abbreviations: GCL represents ganglion cell layer; IPL represents inner plexiform layer; INL represents inner nuclear layer; ONL represents outer nuclear layer; OLM represents outer limiting membrane. The scale bar is equal to 20 µm.

The columnar pattern of BrdU-labeled nuclei across the retina suggests that both nuclei at this stage occur within the same Müller cell, although it is difficult to be certain from the confocal two-dimensional projection images in Figure 1 (C-E). However, by processing the z-stack of images using bioView software (version 1.1.18, free download from the Center for bio-image informatics, UCSB, Santa Barbara, CA) to provide varying contrasts, it appears likely that the vertical pairs of BrdU-labeled nuclei are indeed associated with a single Müller cell “stalk” of intermediate filaments (Figure 2A,B). Figure 2A shows a pair of nuclei lined up directly along one distinct column of intermediate filaments, presumably within a single Müller cell, whereas the image in Figure 2B is rotated slightly so that the lower nucleus in the INL appears off to the side of the major intermediate filament bundle.

**Figure 3:**
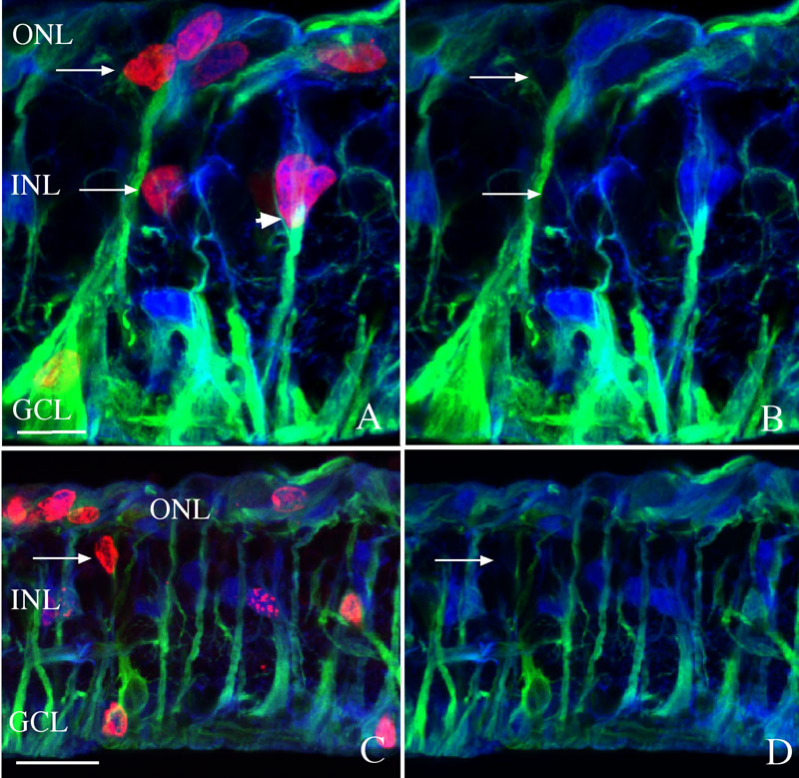
Laser scanning confocal images of rabbit retinas detached for 3 weeks and labeled with anti-bromodeoxyuridine (BrdU; red), anti-vimentin (green), and anti-S100 (blue). BrdU was injected intravitreally on day 3. Many anti-BrdU-labeled nuclei are present throughout the retina, some of which are co-labeled with anti-S100, giving them a purple appearance, while many others do not label with anti-S100 and appear red (arrows). The areas labeled with all three antibodies appear white (**A**, arrowhead). The images in **B** and **D** are the same images as **A** and **C** without the red (anti-BrdU) channel. Abbreviations: GCL represents ganglion cell layer; INL represents inner nuclear layer; ONL represents outer nuclear layer. The scale bars are equal to 50 µm.

Twenty-one days after detachment, many of the anti-BrdU-labeled nuclei in regions away from a subretinal scar appeared unassociated with anti-vimentin-labeled cells (Figure 3A-D, arrows; day 21), especially when viewed through the z-series of images. Some of the nuclei could also be observed in the ganglion cell layer (GCL) at this time (Figure 3C). Vimentin, however, is a cytoskeletal protein and hence does not necessarily fill the entire cell. Therefore, anti-S100, a calcium-binding protein, was used to label the cytoplasm of Müller cells. Since anti-S100 labels the entire cytoplasm with heavy labeling of the cell body, any anti-BrdU-labeled nuclei in S100 positive cells should reflect the overlay of the two fluorochromes (in this case, red+blue=pink). Interestingly, many BrdU-labeled nuclei do not appear to lie within anti-S100-labeled cells (Figure 3A-D, arrows), i.e., the nuclei appear red with no overlying blue or green labeling. By turning off the BrdU (red) channel, it became apparent that there was little to no S100 or vimentin in the cells associated with this subpopulation of BrdU-positive nuclei, although they always lie adjacent to areas of heavy anti-S100 or anti-vimentin labeling (Figure 3B,D, arrows). In an area where the band of intermediate filaments overlays the nucleus of a BrdU- and S100-positive cell, the resulting triple labeling produces a white signal (Figure 3A; arrowhead).

**Figure 4:**
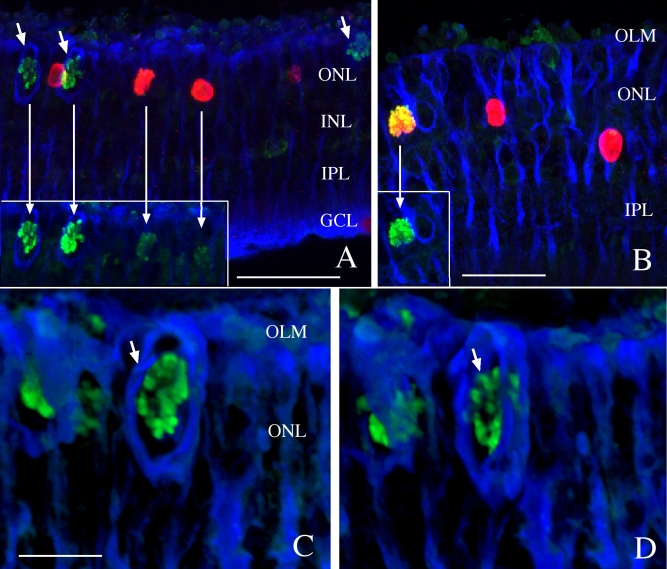
Laser scanning confocal images of rabbit retinas detached for 4 days and labeled with anti-bromodeoxyuridine (BrdU; red), anti-phosphohistone H3 (green), and anti-vimentin (blue). BrdU was injected intravitreally on day 3. Anti-phosphohistone H3 labeling of mitotic cells is only observed in the outer nuclear layer (ONL). Some, but not all, of the anti-phosphohistone H3-labeled cells are also labeled with anti-BrdU (**A**, **B**). The insets in **A** and **B** are cropped from the same image but without the red BrdU channel to more easily visualize the phosphohistone H3 labeling (long arrows point to the same nucleus without the BrdU labeling). Many of the anti-phosphohistone H3-labeled nuclei are surrounded by an accumulation of vimentin filaments (**A**, **C**, **D**, short arrows). Figures **C** and **D** are the same image but digitally rotated by tilting the z-stack in different directions to reveal the three-dimensional architecture of the vimentin filaments around the mitotic cell. The top and bottom images of the z-stack were omitted to visualize the anti-phosphohistone H3 labeling within this region. Abbreviations: INL represents inner nuclear layer; IPL represents inner plexiform layer; GCL represents ganglion cell layer, OLM represents the outer limiting membrane, ONL represents the outer nuclear layer. Scale bars are equal to 50 µm (**A**, **B**) or 20 µm (**C**, **D)**.

To determine if Müller cells were undergoing mitosis in addition to undergoing DNA synthesis in S phase, we labeled sections with both anti-phosphohistone H3 (green) and anti-BrdU (red) at all detachment time points (Figure 4). Many anti-phosphohistone H3-labeled cells were observed, but only at the 4-day time point. Some of these cells were also double labeled with anti-BrdU (Figure 4A,B; insets have the red BrdU channel turned off). Significantly, while anti-BrdU-labeled cells were observed throughout the retina, anti-phosphohistone H3-labeled mitotic figures were observed only in the ONL, suggesting that Müller cell nuclei incorporate BrdU in the INL before migrating to the ONL to divide. The fact that anti-phosphohistone H3-labeled mitotic figures were observed only at 4 days suggests that most of the cell division occurs as a single “wave” within a few days following detachment. Additionally, the anti-phosphohistone H3-labeled cells often appear surrounded by dense anti-vimentin labeling, presumably representing a localized accumulation of intermediate filaments (Figure 4A,C,D, blue, arrows). To appreciate that this “cage” of intermediate filaments completely envelops the mitotic figure formed as the cell divides, Figure 4C,D shows one image tilted slightly in two different directions (anti-phosphohistone H3, green, arrow; anti-vimentin, blue). The top and bottom images of the z-stack were omitted to avoid obscuring the anti-phosphohistone H3 labeling within the cage of vimentin filaments.

### Structural mechanism for nuclear migration

**Figure 5:**
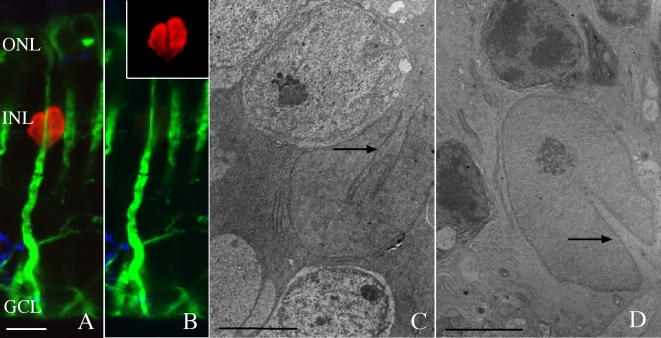
Three images illustrating the indentation or “notch” within a single Muller cell nucleus. **A**, **B**: Laser scanning confocal images of rabbit retina detached for 3 days and labeled with both anti-bromodeoxyuridine (BrdU; red) and anti-vimentin (green). **B** is the same image as shown in **A**, with the two colors separated to better visualize the vimentin filaments (green) and the notch in the (single) nucleus (red, inset). **C**, **D**: Electron micrographs of normal rabbit (**C**) and 50-day detached cat (**D**) retinas showing a notch within Muller cell nuclei (arrows). Note the Müller cell nucleus in **D** now resides among the dark photoreceptor nuclei after detachment. Abbreviations: ONL represents outer nuclear layer; INL represents inner nuclear layer; GCL represents ganglion cell layer. Scale bars equal to 10 µm **A**, **B**; 5 µm **C**, **D**.

**Figure 6:**
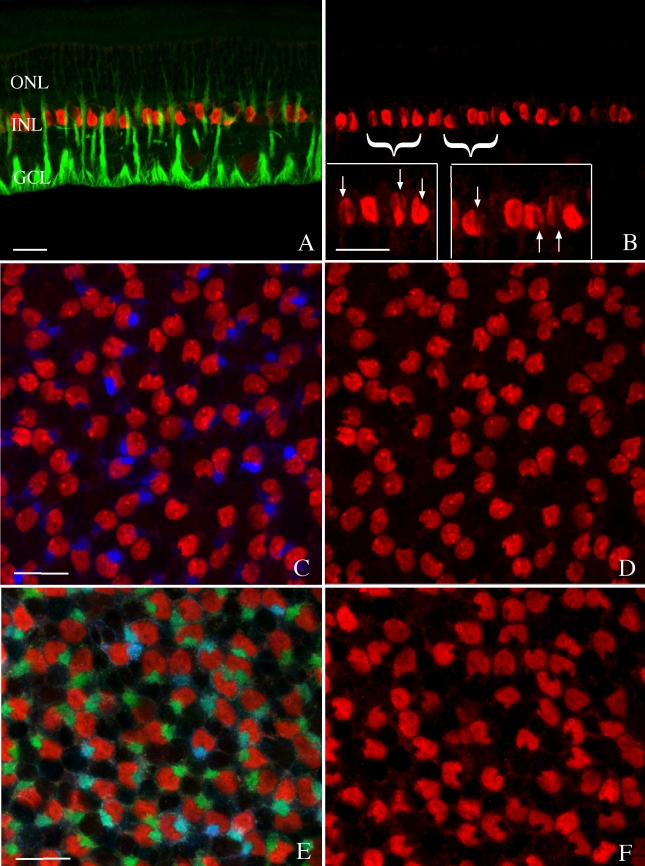
Laser scanning confocal images of radial sections (**A**, **B**) and wholemounts (**C**- **F**) of control rabbit retina labeled with anti-bromodeoxyuridine (BrdU; red), anti-vimentin (green), and anti-glial fibrillary acidic protein (GFAP; blue) from eyes injected with 5-fluorouracil (5-FU). The anti-BrdU detects the 5-FU, which is present in all Müller cell nuclei and illustrates that the notch is visible in most of them (**A**-**F**). In addition, GFAP filaments are present in only some of the notches (**C**, **D**), while vimentin filaments appear to be present in all of the notches (**E**, **F**). The images in the right panels are the same as those on the left but illustrate only the BrdU labeling. The insets in **B** are higher magnifications of the regions marked with the brackets to more easily visualize the notch (arrows). Abbreviations: GCL represents ganglion cell layer; INL represents inner nuclear layer; ONL represents outer nuclear layer. The scale bars are equal to 20 µm.

Since mitotic Müller cells were only observed in the ONL we sought a possible mechanism to explain nuclear migration into the outer retina. In many of the images labeled with anti-BrdU and anti-vimentin, we observed what appeared to be a narrowing of the band of intermediate filaments and their apparent association with a “groove” or “notch” in the Müller cell nucleus (Figure 5A,B). Electron microscopy of tissue from both rabbit and cat retinas clearly demonstrate the deep indentation of the Müller cell nuclei and the presence of intermediate filaments within the notch formed by the indentation (Figure 5C,D, arrows). The prevalence of this nuclear notch and its relationship to the intermediate filament cytoskeleton is demonstrated by confocal imagining of tissue from normal (nondetached) rabbit retinas that had been treated with 5-FU and labeled with anti-BrdU to visualize all Müller cell nuclei. In both radial sections (Figure 6A,B) and wholemount preparations (Figure 6C-F), the notch can be clearly observed in every Müller cell nucleus (red labeling) within a field. In addition, vimentin filaments (green) invariably localize on the side of the cell facing the notch and in many instances can be seen to fill the notch itself. While anti-GFAP labeling also localized to this region (Figure 6C,D, blue), unlike vimentin filaments, GFAP filaments are not present in all the nuclei in the nondetached retina. When present, however, they too bundle with vimentin at the site of the notch.

## Discussion

**Figure 7:**
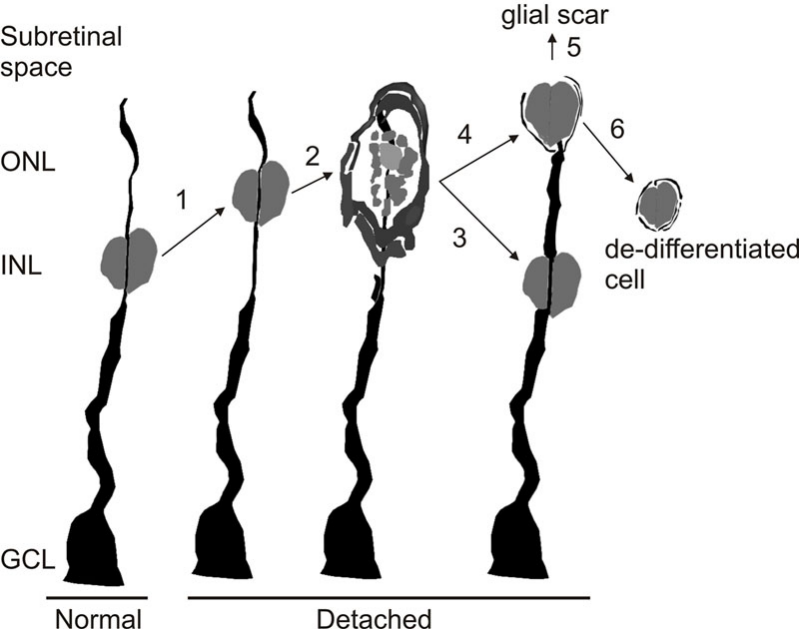
Drawing of a Müller cell with its nucleus and vimentin cytoskeleton showing the proposed hypothesis of how these cells may undergo nuclear division following retinal injury. Step 1, after retinal detachment the nucleus migrates to the outer nuclear layer (ONL); Step 2, vimentin filaments accumulate around the nucleus after which the nucleus undergoes mitosis; Step 3, one nucleus migrates back to the inner nuclear layer (INL); Step 4, the nucleus remaining in the ONL either moves to the subretinal space and contributes to the formation of a glial scar (Step 5) or remains in the retina as a de-differentiated cell (Step 6).

The original goal of this study was to determine if Müller cell proliferation is an important mechanism in the formation of subretinal glial scars. The data, however, provide new information about the reactivity of these highly differentiated glial cells as well as suggest a specific role for their proliferation in glial scar formation. BrdU, injected into the vitreous at the height of the proliferative response following detachment, provided a means to follow the fate of the labeled nuclei over time. Our interpretation of the results is summarized in Figure 7 showing a Müller cell nucleus with its unique structural relationship to the cells’ intermediate filament cytoskeleton (based on the image in Figure 5A). Within 24 h after BrdU injection, anti-BrdU-labeled Müller cell nuclei migrate along the intermediate filament cytoskeleton to the outer retina (Figure 7, step 1). Once there, vimentin filaments assemble around the nucleus, which then undergoes mitosis, as indicated by the anti-phosphohistone H3-labeling data (Figure 7, step 2). Assuming that every BrdU-labeled Müller cell nucleus undergoes division in the outer retina and based on the columnar pattern observed frequently at day 4, the parent Müller cell may occur in a binucleate phase for a short time, about the same time Müller cells begin to grow processes into the subretinal space. Some of these cells may remain binucleate, with one nucleus returning to its normal location in the INL (Figure 7, step 3) and the other nucleus migrating into the processes forming the subretinal scar (Figure 7, steps 4 and 5). Other Müller cells seem to bud off, forming a daughter cell containing one of the nuclei but not labeling with such typical Müller cell markers as anti-vimentin or anti-S100, as if they occur in some stage of dedifferentiation (Figure 7, step 6). Taken together, these data suggest that early Müller cell proliferation is in some way mechanistically coupled to the eventual formation of large subretinal glial scars.

Overall, our observations are remarkably similar to those observed in the injured zebrafish retina [25], although the final outcome is probably very different. Following light damage to the fish retina, which destroys the photoreceptors, Müller cell nuclei migrate to the outer retina where they re-enter the cell cycle. The daughter cell in this case, however, buds off from the parent Müller cell and becomes a retinal progenitor cell, as evidenced by Pax6 expression, which can dedifferentiate and eventually replace lost photoreceptors. A similar phenomenon has also been shown in the chicken retina in response to damage or exogenous growth factors [26]. In this case Müller cells dedifferentiate, proliferate, begin expressing transcription factors normally found in embryonic retinal progenitors, and produce both neurons and glial cells. In the detached rabbit retina, however, we have no evidence that cells in the subretinal space are anything other than Müller cells since they label with typical Müller cell markers. While we have yet to successfully label the BrdU-labeled nuclei that appear in the outer retina with typical markers to progenitor cells as shown in the chicken retina [26], the availability of new progenitor cell markers suitable for use in the mammalian retina in the future will allow addressing this issue.

The observation of a special structural relationship between Müller cell nuclei and the intermediate filament cytoskeleton is also of interest. The observations are certainly suggestive that intermediate filaments may anchor the nuclei in place in the normal retina, provide a “track” along which they migrate in the injured retina, and provide a means of isolating the nuclei in a cage-like structure in the outer retina during mitosis. A similar relationship has been observed between migrating pigment granules and the vimentin cytoskeleton in *Xenopus* melanocytes [27]. It is possible that this cage of filaments isolates the nucleus from the cell and allows for nuclear division without the need for the entire cell to round-up since we found no evidence that these cells retract their inner processes during division.

Some BrdU-labeled nuclei observed here also occur in cells known to participate in other reactions to detachment. For example, a few labeled Müller cell nuclei were observed near the vitreal border in longer term detachments (e.g., 21 days; Figures 3C), and it may be the Müller cells associated with these nuclei that ultimately contribute to epiretinal membranes on the vitreal surface of the retina. In addition, since there are no astrocytes in this region of the rabbit retina (i.e., distant from the medullary rays), the only other cell types that incorporate BrdU are the microglia and macrophages. The robust labeling of these cells with anti-BrdU is more evidence for a significant inflammatory response to retinal detachment [28–30], and while they have been shown to migrate to the photoreceptor layer after detachment [24], their role if any in glial scar formation is unclear.

The data presented here suggest that Müller cell proliferation is a critical step for the formation of glial scars in the retina and that an essential trigger for this scar formation takes place during the period of early proliferation that occurs within a few days of the injury. Clinical trials using anti-proliferative drugs to prevent proliferative diseases in the eye have not proven successful; however, this may be due to the timing of treatment since these drugs are typically given only for a short time during the surgical removal of scar tissue that has already formed, that is, late in the disease state. Anti-proliferative drugs used in prior clinical studies also tend to have a very short half-life in the eye. Two separate studies in this animal model, however, have recently demonstrated that the intravitreal administration, at the time of detachment surgery, of two compounds with a relatively long half-life in the eye effectively reduce both intraretinal proliferation and the formation of subretinal glial scars [31,32]. Those studies support the data presented in this paper that Müller cell proliferation, initiated within a few days after detachment, is critical to the formation of subretinal glial scars.
